# TCGA mRNA Expression Analysis of the Heme Biosynthesis Pathway in Diffusely Infiltrating Gliomas: A Comparison of Typically 5-ALA Fluorescent and Non-Fluorescent Gliomas

**DOI:** 10.3390/cancers12082043

**Published:** 2020-07-24

**Authors:** Mario Mischkulnig, Barbara Kiesel, Daniela Lötsch, Thomas Roetzer, Martin Borkovec, Lisa I. Wadiura, Petra A. Mercea, Florian J. Jaklin, Shawn Hervey-Jumper, Karl Roessler, Mitchel S. Berger, Georg Widhalm, Friedrich Erhart

**Affiliations:** 1 Department of Neurosurgery, Medical University Vienna, 1090 Vienna, Austria; mario.mischkulnig@meduniwien.ac.at (M.M.); barbara.kiesel@meduniwien.ac.at (B.K.); daniela.loetsch-gojo@meduniwien.ac.at (D.L.); martin.borkovec@skyforge.at (M.B.); Lisa.wadiura@meduniwien.ac.at (L.I.W.); petra.mercea@meduniwien.ac.at (P.A.M.); florian.jaklin@students.meduniwien.ac.at (F.J.J.); karl.roessler@meduniwien.ac.at (K.R.); friedrich.erhart@meduniwien.ac.at (F.E.); 2 Comprehensive Cancer Center-Central Nervous System Tumours Unit, Medical University Vienna, 1090 Vienna, Austria; thomas.roetzer@meduniwien.ac.at; 3 Department of Neurology, Division of Neuropathology and Neurochemistry, Medical University Vienna, 1090 Vienna, Austria; 4 Department of Statistics, Ludwig-Maximilians-University, 80539 Munich, Germany; 5 Department of Neurological Surgery, University of California, San Francisco, CA 94143, USA; Shawn.Hervey-Jumper@ucsf.edu (S.H.-J.); Mitchel.Berger@ucsf.edu (M.S.B.)

**Keywords:** 5-ALA, fluorescence, glioma, heme biosynthesis, TCGA, gene expression

## Abstract

5-Aminolevulinic acid (5-ALA) is a fluorescent dye that after metabolization to Protoporphyrin IX (PpIX) by the heme biosynthesis pathway typically leads to visible fluorescence in WHO grade IV but not grade II gliomas. The exact mechanism for high PpIX levels in WHO grade IV gliomas and low PpIX levels in WHO grade II gliomas is not fully clarified. To detect relevant changes in mRNA expression, we performed an in-silico analysis of WHO grade II and IV glioma sequencing datasets provided by The Cancer Genome Atlas (TCGA) to investigate mRNA expression levels of relevant heme biosynthesis genes: Solute Carrier Family 15 Member 1 and 2 (SLC15A1 and SLC15A2), Aminolevulinate-Dehydratase (ALAD), Hydroxymethylbilane-Synthase (HMBS), Uroporphyrinogen-III-Synthase (UROS), Uroporphyrinogen-Decarboxylase (UROD), Coproporphyrinogen-Oxidase (CPOX), Protoporphyrinogen-Oxidase (PPOX), ATP-binding Cassette Subfamily B Member 6 (ABCB6)/G Member 2 (ABCG2) and Ferrochelatase (FECH). Altogether, 258 WHO grade II and 166 WHO grade IV samples were investigated. The mRNA expression levels showed significant differences in 8 of 11 examined genes between WHO grade II and IV gliomas. Significant differences in mRNA expression included increases of HMBS, UROD, FECH and PPOX as well as decreases of SLC15A2, ALAD, UROS and ABCB6 in WHO IV gliomas. Since the majority of changes was found in directions that might actually impair PpIX accumulation in WHO grade IV gliomas, additional studies are needed to analyze the corresponding factors of the heme biosynthesis also on protein level.

## 1. Introduction

Diffusely infiltrating gliomas (DIG) are the most common primary malignant tumors of the central nervous system (CNS) [1]. Depending on distinct histopathological and moleculargenetic characteristics, the WHO differentiates three grades of DIG that are a crucial factor for clinical decision making [2]. While each WHO grade shows specific histopathological features and prognosis, maximal safe resection is the initial therapy of choice in DIG independent of the WHO grade [2,3,4]. Although the extent of resection (EOR) was identified as a key factor for prognosis, precise identification of resection borders still remains challenging due to infiltrative growth of gliomas [5,6,7]. Thus, innovative tools to support intraoperative guidance and detection of residual tumor tissue during surgery of DIG have been established over the past decades to maximize the EOR [8,9,10,11]. Aside from neuronavigation, ultrasound and intraoperative magnetic resonance imaging (MRI), fluorescence-guided surgery using 5-aminolevulinic acid (5-ALA) is a powerful method for improved visualization of high-grade gliomas (HGG; WHO grade III and IV) and is thus nowadays widely used [8,9,10,11]. In contrast to HGG, visible fluorescence is frequently absent in low-grade gliomas (LGG; WHO grade II) [12,13]. However, subvisual 5-ALA fluorescence is present also in LGG based on spectroscopic analyses [13,14,15].

**Figure 1:**
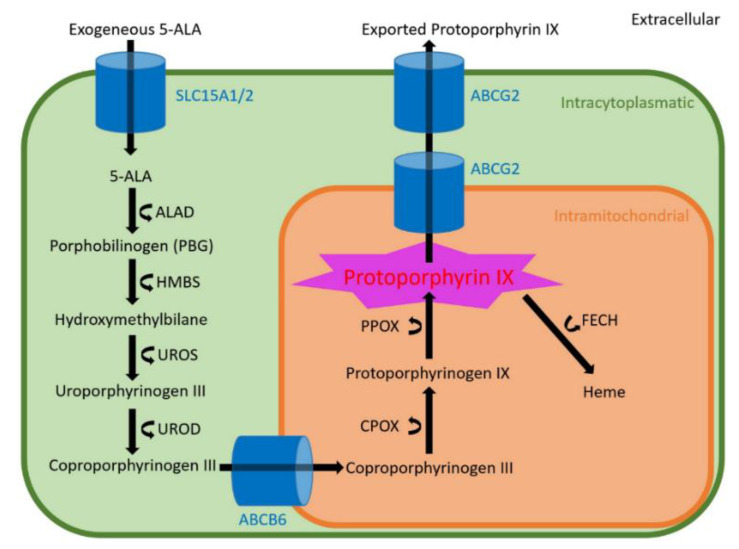
Schematic visualization of the heme biosynthesis pathway with all involved metabolites, transporters (blue) and enzymes (curved arrows).

Routinely, 5-ALA is administered orally approximately 3 h prior to surgery [8,16]. Itself a non-fluorescent agent, 5-ALA is a well-known physiological metabolite in the heme biosynthesis pathway that synthesizes fluorescent Protoporphyrin IX (PpIX), which is the direct precursor of heme [8,17]. Intraoperative 5-ALA induced visualization of DIG is based on PpIX accumulation and is detected by a modified surgical microscope with violet-blue excitation light [8,16]. After intracellular uptake of 5-ALA through Peptide Transporters 1/2 (PEPT1/2; encoding genes: Solute Carrier Family 15 Member 1 and 2—SLC15A1/SLC15A2) it is subject to intracytoplasmatic ALA-Dehydratase (ALAD) assisted metabolization to Porphobilinogen that is deaminated by the Porphobilinogen-Deaminase (PBG-D; encoding gene: Hydroxymethylbilane Synthase; HMBS) [17]. This results in Hydroxymethylbilane, which in turn is transformed to Uroporphyrinogen III by the Uroporphyrinogen-III-Synthase (UROS, encoding gene: UROS) and subsequently decarboxylated by Uroporphyrinogen-Decarboxylase (UROD; encoding gene: UROD) creating Coproporphyrinogen-III [17]. Coproporphyrinogen-III is then transported into mitochondria through the ATP-binding Transporter Cassette Subfamily B Member 6 (ABCB6; encoding gene ABCB6). Initial intramitochondrial metabolization is then facilitated by Coproporphyrinogen-Oxidase (CPOX; encoding gene: CPOX) resulting in Protoporphyrinogen IX and subsequently the active fluorescing metabolite PpIX is created by the Protoporphyrinogen-Oxidase (PPOX; encoding gene: PPOX) [17]. Finally, intracellular depletion of PpIX occurs both through enzymatic catalysis by Ferrochelatase (FECH; encoding gene: FECH), resulting in heme and direct transport into the extracellular space assisted by the ATP-binding transporter Cassette Subfamily G Member 2 (ABCG2; encoding gene: ABCG2) [17]. A schematic visualization of the heme biosynthesis cycle is provided in Figure 1.

While upregulation of the heme biosynthesis pathway is a frequent finding in neoplasms and individual alterations in fluorescent tissue have been described, the exact mechanism resulting in elevated intratumoral PpIX concentration in HGG compared to LGG is still not entirely understood [18,19]. Due to multiple enzymes and transporters being involved in the metabolization of 5-ALA, a variety of alterations in mRNA expression could potentially result in increased intracellular PpIX accumulation [8,17]. A profound understanding of the exact alterations responsible for PpIX accumulation in DIG might ultimately lead to identification of pharmacological targets to further improve the fluorescence effect in LGG and efficiency of photodynamic therapy (PDT).

The aim of our study is thus to provide a comprehensive comparison of mRNA expression of relevant heme biosynthesis factors involved in 5-ALA metabolization between typically fluorescent and non-fluorescent DIG.

## 2. Results

According to our search in the TCGA PANCAN database, we identified a total of 1131 specimens from DIG. Of these, data on mRNA expression was available in 695 specimens. After selection for DIG WHO grade II and GBM (WHO grade IV), 424 specimens formed our primary study cohort and were used for analysis in the logistic regression model. With regard to the WHO tumor grade, our study cohort comprised 258 WHO grade II specimens and 166 WHO grade IV samples. The median age of the included patients was 47 years (IQR 34–60), with a significantly higher median age (median age of 60 years; IQR 51–69) in WHO grade IV compared to WHO grade II patients (median age of 38 years; IQR 30–48; *p* < 0.0005). While men were overall more frequently affected than women (m/f ratio: 1.4), this was more pronounced in GBM (m/f ratio: 2.0) compared to WHO II gliomas (m/f ratio: 1.2). With regard to histopathological subtypes, 65 (25%) WHO grade II tumors were diagnosed as astrocytomas, whereas oligodendrogliomas accounted for 116 (45%) and oligoastrocytomas for 77 (30%) of these tumors. The mean overall survival was significantly longer in WHO grade II gliomas (3515 ± 223 days) compared to WHO grade IV tumors (518 ± 41 days; *p* < 0.0005). Details on patient characteristics are provided in Table 1.

### 2.1. mRNA Expression Model in WHO Grade II vs. IV Gliomas

**Figure 2:**
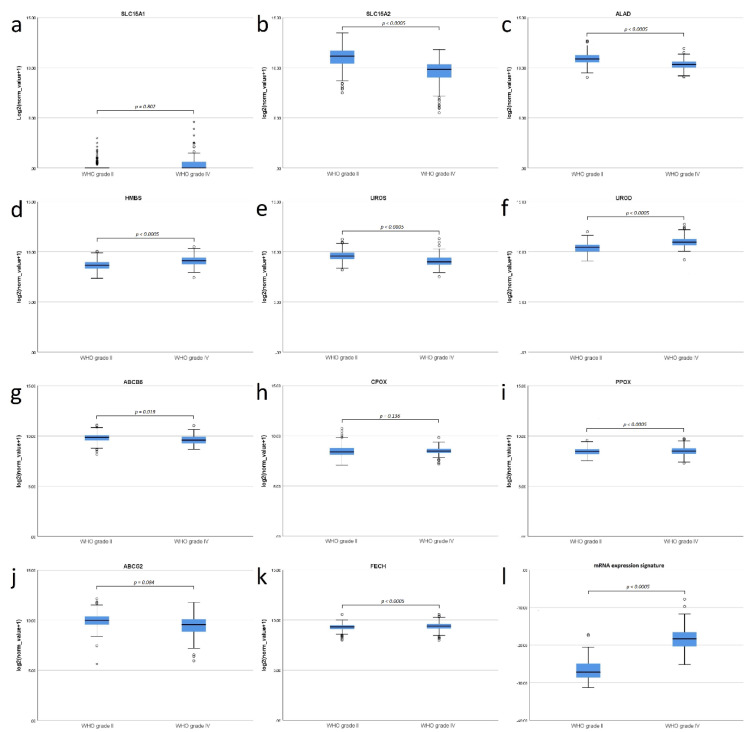
Boxplot diagrams comparing the mRNA expression of relevant factors of the heme biosynthesis pathway between WHO grade II and WHO grade IV gliomas on logarithmic scales. It is of note that an increase of x on the *y*-axis corresponds to an 2x-fold increase of detected copy numbers. (**a**) No significant difference in SLC15A1 mRNA expression. (**b**) Significantly lower SLC15A2 mRNA expression in WHO grade IV gliomas. (**c**) Significantly lower mRNA expression of ALAD in WHO grade IV gliomas. (**d**) Significantly higher HMBS mRNA expression in WHO grade IV gliomas. (**e**) Significantly lower UROS mRNA expression in WHO grade IV gliomas. (**f**) Significantly higher UROD mRNA expression in WHO grade IV gliomas. (**g**) Significantly lower ABCB6 mRNA expression in WHO grade IV gliomas. (**h**) No significant difference in CPOX mRNA expression. (**i**) Significantly lower UROD mRNA expression in WHO grade IV gliomas. (**j**) No significant difference in SLC15A1 expression. (**k**) Significantly higher FECH expression in WHO grade IV gliomas. (**l**) Significantly higher mRNA expression signature in WHO grade IV gliomas compared to WHO grade II gliomas.

Significant differences in mRNA expression between WHO grade II and WHO grade IV gliomas were present for 8 of the 11 examined genes. The greatest difference in mRNA expression was found in SLC15A2 with a 2.66-fold higher mRNA expression in WHO grade II (11.01 ± 1.07) compared to WHO grade IV gliomas (9.60 ± 1.17; *p* < 0.0005). Furthermore, we observed a significant increase of HMBS (9.11 ± 0.53 vs. 8.65 ± 0.50; *p* < 0.0005), UROD (11.01 ± 0.53 vs. 10.37 ± 0.48; *p* < 0.0005), FECH (9.37 ± 0.42 vs. 9.28 ± 0.33; *p* < 0.0005) and PPOX (8.50 ± 0.47 vs. 8.46 ± 0.36; *p* < 0.0005) in WHO grade IV compared to WHO grade II gliomas. Moreover, we found a significant decrease of ALAD (10.33 ± 0.49 vs. 10.92 ± 0.56; *p* < 0.0005), UROS (9.08 ± 0.59 vs. 9.60 ± 0.51; *p* < 0.005) and ABCB6 (9.61 ± 0.45 vs. 9.82 ± 0.43; *p* = 0.019) in WHO grade IV gliomas. In contrast, no statistically significant differences were observed in mRNA expression levels of SLC15A1, CPOX and ABCG2. It is of note that only the changes in HMBS, UROD and PPOX were in directions that might favor increased PpIX accumulation in WHO grade IV gliomas. Results of our main statistical model are shown in Figure 2 and more detailed data are provided in Table 2.

**Figure 3:**
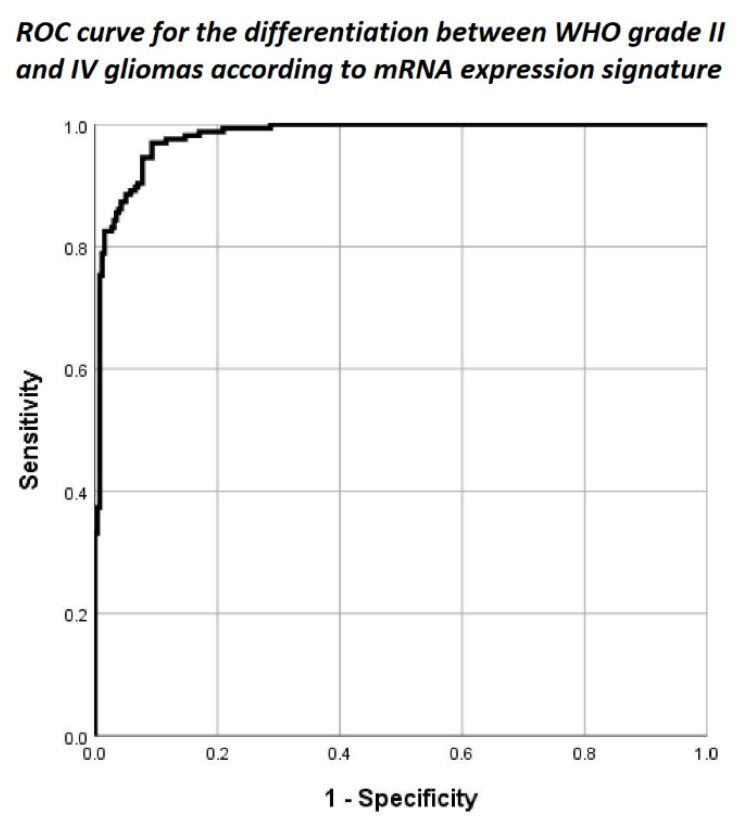
Receiver operating characteristics curve demonstrating an excellent goodness of fit for mRNA expression signature with an area under the curve of 0.981 for the differentiation between WHO grade II and WHO grade IV gliomas.

The overall binary logistic regression model including expression data of all analyzed mRNA levels demonstrated a statistically significant difference in mRNA expression between WHO grade II and WHO grade IV gliomas (*p* < 0.0005; Figure 2). No significant differences in mRNA expression signature between WHO grade II astrocytomas, oligodendrogliomas and oligoastrocytomas were present (*p* = 0.268). To evaluate the performance of our mRNA regression model, we used ROC analysis based on the mRNA expression signature calculated according to the above described formula (Material and Methods, Statistical analysis). According to ROC analysis, we found an overall high predictive power of our model with an area under the curve of 0.981 and maximum diagnostic accuracy for differentiation of WHO grade IV from WHO grade II at a mRNA expression signature of −23.28 with a corresponding sensitivity of 97.0% and specificity of 90.7%. The ROC curve is shown in Figure 3.

### 2.2. Additional Application of Our Regression Model in WHO Grade III Gliomas and Normal Brain Tissue

**Figure 4:**
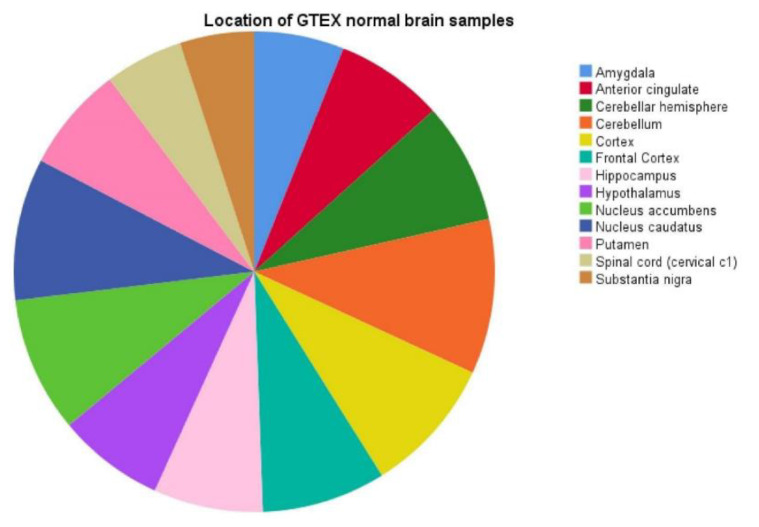
Illustration demonstrating the different locations of normal brain tissue samples from the GTEx database.

Since we observed a significant difference in mRNA expression signature between WHO grade II and WHO grade IV gliomas, we tested our regression model also in WHO grade III gliomas and normal brain tissue. For this purpose, we leveraged the WHO grade III glioma mRNA dataset from the TCGA PANCAN database (*n* = 270) and additionally obtained normal brain tissue mRNA data identified from the GTEx database (*n* = 1141). Details on patient characteristics of the enlarged dataset are provided in Table 1 and the respective location of GTEx normal brain samples are given in Figure 4.

**Figure 5:**
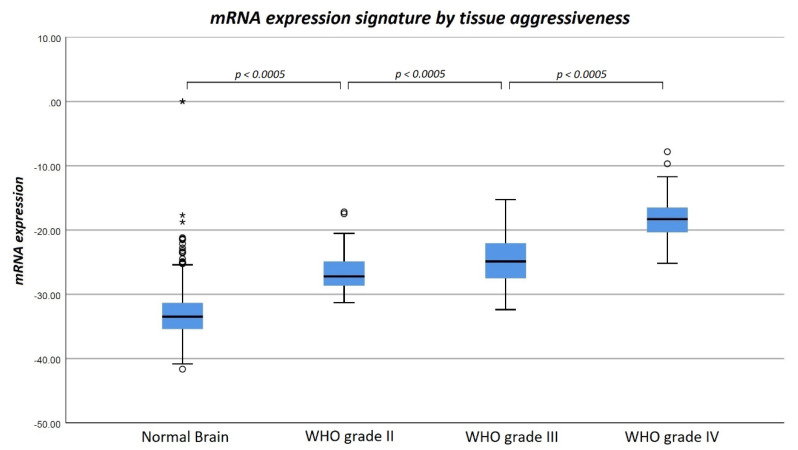
Boxplot diagram of mean mRNA expression signature of WHO grade II–IV gliomas as well as normal brain tissue. It is of note that mRNA expression signature shows a strictly monotonical increase with tissue aggressiveness. The observed differences were statistically significant between all subgroups.

Additional application of our model also in WHO grade III gliomas and normal brain tissue resulted in the observation of a strictly monotonical increase in mRNA expression signature with tissue aggressiveness (normal brain tissue: −33.02 ± 3.91, WHO grade II glioma: −26.72 ± 2.58, WHO grade III glioma: −24.56 ± 3.53 and WHO grade VI glioma: −18.31 ± 3.04). The observed differences were statistically significant between all subgroups (*p* < 0.0005). A boxplot diagram of the mRNA expression signature in all histopathological WHO grades and normal brain tissue is shown in Figure 5.

## 3. Discussion

Over the past 20 years, 5-ALA fluorescence-guided surgery has been established as valuable visualization technique during resection of HGG with detectable fluorescence in almost all of these tumors [16,20]. As a consequence, a significantly higher rate of complete resections and prolonged progression-free survival was found in patients suffering from WHO grade IV gliomas after 5-ALA fluorescence-guided resection [16]. In contrast, visible 5-ALA fluorescence is only rarely detectable in WHO grade II gliomas and thus this technique is usually unable to improve the EOR in such tumors [12,21]. Initially, a breakdown of the blood brain barrier (BBB) resulting in distinct leakage of contrast-enhancement (CE) on MRI was believed to be essential for presence of visible 5-ALA fluorescence [20,22]. However, visible 5-ALA fluorescence was recently also observed in gliomas with non-significant CE [13,23]. Thus, further key factors seem to be responsible for presence of visible 5-ALA fluorescence aside from a breakdown of the BBB. Since 5-ALA is physiologically metabolized to fluorescing PpIX by specific enzymes or transporters of the heme biosynthesis, it is likely that this pathway plays a key role for presence or absence of visible 5-ALA fluorescence in brain tumors [8,19,24]. Prior studies investigating specific heme biosynthesis factors in relation to 5-ALA fluorescence were mainly based on cell culture analyses and examined a comparatively small number of specimens [19,25]. Furthermore, recent analyses did not find significant differences in mRNA expression of heme biosynthesis factors, whereas an earlier publication demonstrating a distinct upregulation in fluorescent samples had to be retracted due fundamental technical issues [19,25,26]. The aim of this study was thus to perform a comprehensive analysis of a large dataset provided by the TCGA examining the gene expression of relevant factors involved in the metabolization of 5-ALA suitable to detect even minor differences in mRNA expression between typically fluorescent WHO grade IV and non-fluorescent WHO grade II gliomas.

### 3.1. Differences in mRNA Expression

In this study, significant differences in mRNA expression between WHO grade II and WHO grade IV gliomas were found in the majority (8 of 11) of analyzed genes. Interestingly, however, in only three genes mRNA expression (HMBS, UROD and PPOX) was altered in the direction expected to result in increased PpIX concentrations in WHO grade IV gliomas, whereas the remaining 5 up-/downregulations were actually observed in the respective opposite directions. The strongest fold-wise effect of these plausible seeming alterations was a 1.56-fold increase in UROD in WHO grade IV compared WHO grade II gliomas. While the increase in mRNA levels of PpIX synthesizing UROD, HMBS and PPOX in WHO grade IV samples seem credible according to the role of the respective gene products in the heme biosynthesis pathway, such alterations have not been described before.

Surprisingly, most statistically significant alterations in mRNA gene expression were observed in a direction that, if passed on to the protein level accordingly, should actually result in lower levels of intracellular PpIX in WHO grade IV gliomas. These alterations included downregulations of a gene responsible for cellular uptake of 5-ALA (SLC15A2) and transport of its metabolites into mitochondria (ABCB6) as well as specific genes coding for enzymes of the heme biosynthesis pathway (ALAD and UROS) in WHO grade IV tumors. In addition, higher mRNA levels of the PpIX degrading protein FECH were observed in WHO grade IV gliomas. This finding is especially interesting, since an earlier smaller study contrarily found lower FECH mRNA expression levels in GBM (*n* = 41) compared to LGG (*n* = 17) and normal brain tissue (*n* = 15) [27]. While in multiple studies [19,25] comparing heme biosynthesis factors in gliomas (one of them meanwhile retracted) no significant FECH up- or downregulations were described, and the role of FECH in naturally occurring 5-ALA induced fluorescence thus remains unclear, there is convincing evidence that artificial inhibition of this enzyme actually results in higher PpIX levels due to decreased degradation [26,27,28].

For SLC15A1, CPOX and ABCG2 mRNA expression, no differences between typically fluorescent WHO grade IV and non-fluorescent WHO grade II gliomas were detected. While ABCG2 downregulation was described in individual HGG cell lines [19,29], no significant alterations were observed in the investigation available for either ABCG2 nor SLC15A2. CPOX is the gene previously most controversially studied in fluorescent vs. non-fluorescent gliomas. While Takahasi et al. found markedly higher levels of CPOX mRNA expression in fluorescent tumors, this study from 2011 was finally retracted [26]. In a more recent study from 2017, Pustogarov et al. even reported a significant decrease in mRNA concentrations of this gene in fluorescence-positive glioma cell cultures compared to non-fluorescing glioma cells [19]. It is thus of note that in contrast to the documented alterations in CPOX mRNA expression in both directions for verified fluorescent cell lines, our study found an almost identical expression in WHO grade II and IV gliomas.

Altogether, due to the high number of examined specimens, our comprehensive investigation was successful in identifying differences in mRNA expression between WHO grade II and WHO grade IV gliomas. It is of note, however, that most of these alterations were comparatively mild, and most changes were observed in directions contrary to what might be expected based on the respective typical fluorescence behavior of these tumors. Thus, the findings of our present study are surprising. An important hint for their interpretation may be provided by the work of Pustogarov et al. [19]. As described above, this study found decreased levels of CPOX mRNA expression in fluorescent tumors with PCR analyses; however, the corresponding CPOX protein showed significantly elevated concentrations [19]. Therefore, the authors considered the presence of alternative splice variants as the most likely explanation for this paradoxical finding based on mRNA expression [19]. Since the majority of changes in our study were observed in directions that might actually impair PpIX accumulation in WHO grade IV gliomas, we hypothesize that phenomena such as alternative splicing and post-transcriptional modifications might play a crucial role in 5-ALA metabolization in gliomas. Consequently, the data in the literature and our own study demonstrate the complexity of alterations leading to detectable 5-ALA induced fluorescence. Our findings also indicate that future studies are needed to comprehensively investigate not only relevant changes in mRNA expression, but also on the resulting protein level.

The validity of the observed mRNA differences between WHO grade II and IV gliomas was further verified by application of our logistic regression model to two additional, independent datasets (WHO grade III gliomas and normal brain tissue). In accordance with our initial observations in the main study cohort, we found mRNA expression signature in WHO grade III gliomas fitting between WHO grade II and WHO IV gliomas. Furthermore, mRNA expression signature in normal brain tissue samples was markedly lower than in glioma samples of all WHO grades.

### 3.2. Additional Factors Influencing 5-ALA Induced PpIX Accumulation

Aside from the WHO grade, additional factors might impact the 5-ALA induced PpIX accumulation in gliomas. (1) In this sense, 5-ALA fluorescence might also be associated with distinct subtypes according to the Verhaak classification [30,31]. (2) Another aspect consists of histopathological parameters such as cellularity, MIB-LI and neovascularization that have previously been shown to correlate with 5-ALA fluorescence [32]. (3) In addition, the tumor type (astrocytoma/oligoastrocytoma/oligodendroglioma) within the subgroup of WHO grade II gliomas might also influence the 5-ALA induced PpIX accumulation. However, our statistical analysis showed no significant differences in the mRNA expression signature between the distinct tumor types. Based on these observations we thus did not find evidence for a substantial impact of the tumor types in WHO grade II gliomas. (4) Moreover, a current study demonstrated that highly glycolytic tumors do require less heme [33]. This mechanism might result in heterogeneity of the metabolic composition of especially high-grade gliomas. (5) Finally, it is well known that the blood–brain barrier plays a considerable role in the 5-ALA induced PpIX accumulation aside from metabolic alterations within the heme biosynthesis pathway [34]. Taken together, all these potentially relevant factors should be considered and investigated in future studies to fully elucidate the complex landscape of 5-ALA biology.

### 3.3. Clinical Relevance

Identification of the exact mechanisms responsible for 5-ALA fluorescence might facilitate the pharmacological modulation of the fluorescence effect in the future. First pharmacological inhibitors of specific heme biosynthesis genes such as N-methylmesoporphyrin and Griseofulvin (both Inhibitors of FECH) have been identified [35,36]. Furthermore, RNA interference (RNAi) offers great potential as a novel therapeutic strategy to specifically and efficiently silence further target genes of the heme biosynthesis pathway [37]. As a consequence, we expect that WHO grade II gliomas might be visualized during surgery by visible 5-ALA fluorescence in the future, and thus the EOR might be significantly improved, similar to WHO grade IV gliomas. Additionally, we expect to markedly increase the efficiency of PDT with resulting maximized tumor cell death while concurrently preserving healthy brain tissue and thus neurological function.

### 3.4. Limitations

Even though our study provides a comprehensive overview of mRNA expression data from a large number of WHO grade II and WHO grade IV gliomas, important limitations have to be considered: (1) Since no data on 5-ALA use and status are available in the TCGA dataset, we did not directly compare fluorescent vs. non-fluorescent samples but used WHO grade II vs. WHO grade IV as closely related surrogate subgroups. In this sense, only single cases of WHO grade IV with lack of 5-ALA fluorescence were reported [18,38]. Additionally, 5-ALA fluorescence is absent in the vast majority of WHO grade II gliomas (>90%) [20]. Although the tumor grade is not perfectly identical with the 5-ALA fluorescence status, it is still a justifiable and sufficient approximation for it. Nevertheless, subsequent studies could specifically focus on this aspect and explicitly evaluate heme metabolization factors in relation to 5-ALA fluorescence status independent of tumor grade. (2) No information on distinct molecular markers such as 1p/19q co-deletion was available in the TCGA dataset analyzed. Therefore, we could only rule out a relevant effect of WHO grade II glioma subtype (astrocytoma/oligoastrocytoma/oligodendroglioma) on the mRNA expression signature, but no definitive conclusion on the role of molecular 1p/19q co-deletion could be drawn at this time. Future studies analyzing heme biosynthesis in the context of 5-ALA should thus specifically consider molecular tumor markers like 1p/19q co-deletion.

## 4. Materials and Methods

In this study, we conducted a systematic analysis of mRNA expression levels of factors responsible for cellular uptake of 5-ALA, intracellular metabolization through the heme biosynthesis pathway and degradation of the resulting fluorescent PpIX. The investigation of the role of heme biosynthesis factors for 5-ALA fluorescence was approved by the ethics committee of the Medical University Vienna. Our study was based on data of the Cancer Genome Atlas (TCGA) provided by the National Cancer Institute and Human Genome Research Institute. Data were accessed using the freely available online tool Xena provided by the Genomics Institute at the University of California Santa Cruz [39]. Since no data directly on the intraoperative 5-ALA fluorescence status were available, we selected typically fluorescent WHO grade IV glioblastomas (GBM) and characteristically non-fluorescent WHO grade II diffuse gliomas for our analyses [30,40]. The TCGA Pan-Cancer (PANCAN) was identified as the most comprehensive dataset available in regard to DIG and was thus used for our study. Within the TCGA PANCAN dataset, entries were initially filtered for histopathological diagnosis of DIG (astrocytoma, oligodendroglioma, oligoastrocytoma or GBM). Subsequently, GBM (WHO grade IV) and WHO grade II gliomas were selected and normalized mRNA expression data (in “log2(norm_value + 1)” format) were collected for the following genes [encoded protein]: SLC15A1 [PEPT1], SLC15A2 [PEPT2], ABCB6 [ABCB6], ALAD [ALAD], HMBS [PBG-D], UROS [UROS], UROD [UROD], CPOX [CPOX], PPOX [PPOX], ABCG2 [ABCG2] and FECH [FECH]. Datasets of samples without mRNA expression data were subsequently excluded. For additional testing of our mRNA expression analysis method used for WHO grade II and IV gliomas, in a second step, we investigated mRNA expression also in WHO grade III gliomas and normal brain tissue. For this purpose, we additionally collected mRNA expression data from WHO grade III gliomas identified from the TCGA PANCAN database as well as from normal brain tissue samples obtained from the Genotype-Tissue Expression (GTEx) Program of the National Institutes of Health [41,42]. For the current study, demographic patient data were collected including patient age, gender and survival status.

### Statistical Analysis

Statistical analyses and figure production were conducted using SPSS statistical software (Version 25.0, SPSS Inc.). Descriptive analysis included median patient age, gender distribution, histopathological tumor subtypes and WHO grades. For visualization of mRNA expression, separate boxplot diagrams for all analyzed genes were created. Furthermore, inferential statistical analysis of mRNA expression was performed using a logistic regression model incorporating mRNA expression data of all 11 analyzed genes. Furthermore, a statistical model of the mRNA expression in all examined genes was established according to the logit coefficients (B) of the regression model in WHO grade II/IV gliomas:

mRNA expression signature = B^SLC15A1^ × log_2_(norm_value + 1)^SLC15A1^ + B^SLC1A2^ × log_2_(norm_value + 1)^SLC15A2^ + B^ALAD^ × log_2_(norm_value + 1)^ALAD^ + B^HMBS^ × log_2_(norm_value + 1)^HMBS^ + B^UROS^ × log_2_(norm_value + 1)^UROS^ + B^UROD^ × log_2_(norm_value + 1)^UROD^ + B^ABCB6^ × log_2_(norm_value + 1)^ABCB6^ + B^CPOX^ × log_2_(norm_value + 1)^CPOX^ + B^PPOX^ × log_2_(norm_value + 1)^PPOX^ + B^ABCG2^ × log_2_(norm_value + 1)^ABCG^ + B^FECH^ × log_2_(norm_value + 1)^FECH^ (1)

To test for goodness of fit, a receiver operating characteristics (ROC) analysis was conducted for the predictive accuracy of this expression regarding WHO grades II and IV. Since all subtypes of WHO grade II gliomas (astrocytoma/oligodendroglioma/oligoastrocytoma) were included in our investigation, differences between these subgroups were analyzed to test for a possible bias on our main analysis. For further testing of our logistic regression model, calculations based on this model were also performed in WHO grade III gliomas and normal brain tissue that were not included in the initial analysis. Testing for differences in mRNA expression signature between these histological subgroups to validate our main statistical model was performed using ANOVA and subsequent unpaired *t*-tests between distinct subgroups. Margin of statistical significance was set at *p* < 0.05, correction for multiple testing was performed by inclusion of all analyzed enzymes/transporters of heme biosynthesis in a single regression model.

## 5. Conclusions

In this study, we conducted a comprehensive, TCGA-based in silico analysis of relevant mRNA expression levels related to the metabolization of 5-ALA in typically fluorescent WHO IV gliomas versus typically non-fluorescent WHO II gliomas. Therewith, we close a relevant knowledge gap: Even though TCGA datasets have been extensively used for glioma research, no prior study aimed at gene expression differences with special focus on the 5-ALA fluorescence behavior. According to our data, we demonstrate significant differences in mRNA expression in 8 of 11 examined genes between WHO grade II and IV gliomas in the largest cohort analyzed in this respect so far. Since some of these alterations in mRNA expression are contrary to what might be expected-based our current understanding of 5-ALA fluorescence behavior, additional studies are needed to analyze the corresponding factors of the heme biosynthesis pathway also on protein level.

## Figures and Tables

**Table 1:** Patient Characteristics.

Characteristic		Main Study Cohort						Extended Study Cohort					
		Overall		WHO Grade II		WHO Grade IV		Overall		Normal Brain Tissue		WHO Grade III	
		*n*	(%)	*n*	(%)	*n*	(%)	*n*	(%)	*n*	(%)	*n*	(%)
**Number of patients**		424	(100)	258	(100)	166	(100)	1411	(100)	1141	(100)	270	(100)
**Age**	**median (range)**	47 (34–60)		38 (30–48)		60 (51–69)		N/A		N/A		44 (34–56)	
**Gender**	**(male:female)**	1.4:1		1.2:1		1.8:1		2.0:1		2.2:1		1.3:1	
**Histological subtype**													
	**no tumor**	-	-	-	-	-	-	1141	(81)	1141	(100)	-	-
	**astrocytoma**	65	(15)	65	(25)	-	-	132	(9)	-	-	132	(49)
	**oligodendroglioma**	116	(28)	116	(45)	-	-	82	(6)	-	-	82	(30)
	**oligoastrocytoma**	77	(18)	77	(30)	-	-	56	(4)	-	-	56	(21)
	**glioblastoma**	166	(39)	-	-	166	(100)	-	-	-	-	-	-

**Table 2:** mRNA Expression Data.

mRNA	WHO °II		WHO °IV		Logit Coefficient (B)	*p*	Exp (B)	95% Confidence Interval for Exp (B)	
	Mean	SD	Mean	SD				Lower Bound	Upper Bound
SLC15A1	0.19	0.42	0.40	0.75	0.11	0.80	1.11	0.48	2.58
SLC15A2	11.01	1.07	9.60	1.17	−1.26	0.00	0.29	0.17	0.47
ALAD	10.92	0.56	10.33	0.49	−2.22	0.00	0.11	0.04	0.29
HMBS	8.65	0.50	9.11	0.53	2.63	0.00	13.93	3.52	55.15
UROS	9.60	0.51	9.08	0.59	−2.93	0.00	0.05	0.02	0.13
UROD	10.37	0.48	11.01	0.53	2.99	0.00	19.84	6.60	59.64
ABCB6	9.82	0.43	9.61	0.45	−1.43	0.02	0.24	0.07	0.79
CPOX	8.49	0.61	8.50	0.36	0.66	0.14	1.93	0.81	4.59
PPOX	8.46	0.36	8.50	0.47	−2.86	0.00	0.06	0.01	0.26
ABCG2	9.98	0.71	9.44	1.07	−0.48	0.08	0.62	0.36	1.07
FECH	9.28	0.33	9.37	0.42	2.49	0.00	12.04	3.09	46.99
